# *The Following Letter from* Dr. Marshall *Has Been Transmitted to the Editors for Insertion in the Medical and Physical Journal*

**Published:** 1801-03

**Authors:** W. Marshall

**Affiliations:** His Majesty's Ship, Foudroyant. Mahon


*The following Letter from* Dr. MARSHALL *has been transmitted to the Editors for Insertion in the Medical and Physical Journal.*

---

DEAR FRIEND,

FROM Tetuan Bay I did myſelf the pleaſure of writing you, informing you that I was buſily employed inoculating the fleet under Lord Keith, who iſſued a general order for that purpoſe to all the ſhips under his command; ſince then I have continued with him, and ſhall proceed up to Malta, for which place we ſet ſail to-morrow.

I have now to tell you, what before I was ſuſpicious of, that the matter I brought with me from England, after being kept three months, became quite inert; and though it was capable of producing an inflammation in the inoculated part, yet the puſtule produced, did not partake in the leaſt of the Cow-pox characteriſtic marks, nor produced any conſtitutional effect whatever; accordingly, all thoſe upon whom it was tried, I again inoculated with the recent fluid matter. I have alſo, in preſence of the medical men of Mahon, the jurats, and a number of the inhabitants, inoculated an infant who had had the the Cow-pox, with the Small-pox virus, procured by one of the phyficians prefent; the child refifted the infection, and of courfe, convinced thofe who were unbelievers of the efficacy of the Cow-pox. Thus, the bleffing of the Vaccine Inoculation is every day fpreading more and more: I hope, in a very fhort time, to carry it up to Sicily, along with the fleet, where it will have a more extenfive field to range in.

The army, from the liberal ufe of frefh provifions and fruit, have got free from the fcurvy; but thofe regiments that were recruited out of the militia, are falling off very faft, numbers of them dying daily: I think, upon their landing in Egypt, they will mufter very thin. We have certain accounts of fix veffels having failed from Toulon on the 13th inft. with troops (fuppofed about 6,000) and ftores for Alexandria; if they efcape our fhips and land fafe, it may ferve to embarrafs our operations there very much. I have had an opportunity of perufing many papers and documents refpecting the plague; and from what I there obferve, I fhould not hefitate in going with the expedition, for the purpofe of procuring better information upon the fubject, had I not another material object to employ me. It will give me great pleafure to hear from you when you have leifure. Believe me, &c.

*His Majesty's Ship, Foudroyant.* W. MARSHALL.

